# Osteocalcin modulates parathyroid cell function in human parathyroid tumors

**DOI:** 10.3389/fendo.2023.1129930

**Published:** 2023-03-30

**Authors:** Chiara Verdelli, Giulia Stefania Tavanti, Irene Forno, Valentina Vaira, Riccardo Maggiore, Leonardo Vicentini, Paolo Dalino Ciaramella, Francesca Perticone, Giovanni Lombardi, Sabrina Corbetta

**Affiliations:** ^1^ Laboratory of Experimental Endocrinology, IRCCS Istituto Ortopedico Galeazzi, Milan, Italy; ^2^ Department of Biomedical, Surgical and Dental Sciences, University of Milan, Milan, Italy; ^3^ Division of Pathology, Fondazione IRCCS Ca` Granda Ospedale Maggiore Policlinico, Milan, Italy; ^4^ Department of Pathophysiology and Transplantation, University of Milan, Milan, Italy; ^5^ Endocrine Surgery, IRCCS Ospedale San Raffaele, Milan, Italy; ^6^ Endocrine Surgery, IRCCS Istituto Auxologico, Milan, Italy; ^7^ Endocrinology Unit, ASST Grande Ospedale Metropolitano Niguarda, Milan, Italy; ^8^ Endocrinology Unit, IRCCS Ospedale San Raffaele, Milan, Italy; ^9^ Laboratory of Experimental Biochemistry and Molecular Biology, IRCCS Istituto Ortopedico Galeazzi, Milan, Italy; ^10^ Department of Athletics, Strenght and Conditioning, Poznań University of Physical Education, Poznań, Poland; ^11^ Endocrinology and Diabetology Service, IRCCS Istituto Ortopedico Galeazzi, Milan, Italy

**Keywords:** parathyroid tumor, osteocalcin, CASR, GPRC6A, ERK, beta-catenin

## Abstract

**Introduction:**

The bone matrix protein osteocalcin (OC), secreted by osteoblasts, displays endocrine effects. We tested the hypothesis that OC modulates parathyroid tumor cell function.

**Methods:**

Primary cell cultures derived from parathyroid adenomas (PAds) and HEK293 cells transiently transfected with the putative OC receptor GPRC6A or the calcium sensing receptor (CASR) were used as experimental models to investigate γ-carboxylated OC (GlaOC) or uncarboxylated OC (GluOC) modulation of intracellular signaling.

**Results:**

In primary cell cultures derived from PAds, incubation with GlaOC or GluOC modulated intracellular signaling, inhibiting pERK/ERK and increasing active β-catenin levels. GlaOC increased the expression of *PTH, CCND1* and *CASR*, and reduced *CDKN1B/p27* and *TP73*. GluOC stimulated transcription of *PTH*, and inhibited *MEN1* expression. Moreover, GlaOC and GluOC reduced staurosporin-induced caspase 3/7 activity. The putative OC receptor GPRC6A was detected in normal and tumor parathyroids at membrane or cytoplasmic level in cells scattered throughout the parenchyma. In PAds, the membrane expression levels of GPRC6A and its closest homolog CASR positively correlated; GPRC6A protein levels positively correlated with circulating ionized and total calcium, and PTH levels of the patients harboring the analyzed PAds. Using HEK293A transiently transfected with either GPRC6A or CASR, and PAds-derived cells silenced for *CASR*, we showed that GlaOC and GluOC modulated pERK/ERK and active β-catenin mainly through CASR activation.

**Conclusion:**

Parathyroid gland emerges as a novel target of the bone secreted hormone osteocalcin, which may modulate tumor parathyroid CASR sensitivity and parathyroid cell apoptosis.

## Introduction

Primary hyperparathyroidism (PHPT) is one of the most common endocrine disorder, and it is characterized by inappropriate secretion of PTH from parathyroid tumors and hypercalcemia (1). PHPT is one of the main causes of secondary osteoporosis, with a prevalence of 1 at 1000 in postmenopausal women (1). It is often associated with high bone turnover (2); high bone turnover induces the release of a number of bioactive molecules from bone, among which osteocalcin (OC) is thought to play extraskeletal endocrine functions. OC is a small non-collagenous protein mainly produced by osteoblasts and is highly represented in bones of most vertebrates. Human OC contains up to three highly conserved gamma-carboxyglutamic acid residues (GlaOC), at positions 17, 21 and 24 (3), which are thought to increase calcium-binding strength, improving mechanical properties of the bone matrix. Recent studies *in vitro* and in animal models revealed that OC may exert also important endocrine functions, affecting energy metabolism and male fertility (4). The endocrine effects seem to be mediated by the uncarboxylated form of OC (GluOC) (5), while data regarding the carboxylation state-related function of the active form of OC are controversial (6, 7).

Tumors of the parathyroid glands are common, mostly benign, though sometimes associated with severe and life-threatening PHPT. Parathyroid tumors are heterogeneous in the severity of PTH secretion, cell proliferation, and genetic background (8). It is known that persistent secondary hyperparathyroidism, such as that induced by idiopathic hypercalciuria or malabsorption-related vitamin D deficiency, may stimulate parathyroid cell proliferation and autonomous PTH hypersecretion (9).

Therefore, it may be conceived that increased bone turnover and consequent increased OC release from bone might promote parathyroid cell proliferation and/or modulate PTH secretion. In line with this hypothesis, both circulating GlaOC and GluOC levels have been found to be increased in PHPT patients (10–12), and elevated levels of circulating OC have been suggested to be predictors of multiglandular disease in PHPT patients (13). Indeed, a direct effect of OC on parathyroid cell function has never been investigated until now.

GPRC6A, a widely expressed G-protein coupled receptor, is proposed to be the putative receptor of OC and a master regulator of complex endocrine networks and metabolic processes (14, 15). GPRC6A is the closest mammalian homolog of the calcium-sensing receptor (CASR), which is the molecular mechanism inhibiting PTH secretion and parathyroid cell proliferation. CASR is the target of the currently available drugs cinacalcet and etelcalcetide for the control of hyperparathyroid diseases (16). Both CASR and GPCR6A are often modulated by the same orthosteric and allosteric ligands and have overlapping expression patterns (17). It is thus conceivable that the receptors could either directly form heterodimers or remain expressed as homodimers in the same cell, having different effects on intracellular signaling.

The present study aims to investigate: 1) the effects of GlaOC and GluOC on the modulation of intracellular signaling pathways and of the parathyroid specific genes expression in human parathyroid tumor cells, 2) the expression of GPRC6A in parathyroid adenomas-derived cells, 3) the distinct effects of the stimulation with GlaOC or GluOC in HEK293A cells transfected with GPRC6A or CASR, and 4) the effects of GlaOC and GluOC on parathyroid cell apoptosis.

## Materials and methods

### Parathyroid tissue samples

Fresh samples from 45 parathyroid adenomas (PAds) were collected immediately after surgical removal from patients with a diagnosis of PHPT, caused by a single parathyroid adenoma, partly snap frozen and partly dissociated for primary cell cultures. In all PHPT patients, fasting plasma ionized calcium, serum total calcium, and PTH were routinely measured to diagnose PHPT. This study was approved by the Institutional Ethical Committee (Ospedale San Raffaele Ethical Committee, protocol no. GPRC6A PARA, 07/03/2019; CE40/2019), and informed consent was obtained from all patients.

### Cell cultures

Samples from PAds were cut into 1 mm^3^ fragments, washed with PBS and partially digested with 2 mg/mL collagenase type I (Worthington, Lakewood, NJ, USA) for 90 minutes. Digested tissues were filtered with a cell strainer (100 μm Nylon, BD Falcon, Rignano Flaminio, Italy) to obtain a single cell suspension and cultured in DMEM supplemented with 10% fetal bovine serum, 2 mmol/L glutamine and 100 U/mL penicillin-streptomycin.

The human embryonic kidney HEK293A cell line (Catalog n.R705-07, Invitrogen, ThermoFisher Scientific, Carlsbad, CA, USA) was maintained until 30 passages and cultured in the same medium of PAds-derived cells, as described above.

Since a human parathyroid cell line is not commercially available and due to the difficulty in performing a long-term parathyroid primary cell culture, we used PAds-derived cells, two days after isolation, and transiently transfected HEK293A cell line as models for our experiments.

### Treatment of PAds-derived cells with GlaOC and GluOC

Before treatment with GlaOC or GluOC, cells were serum-starved overnight and pretreated for 30 minutes with physiological saline solution PSS (NaCl 125 mM, KCl 4 mM, HEPES 20 mM, D-Glucose 0.1%, NaH_2_PO_4_ 0.8 mM, MgCl_2_ 1 mM, pH 7.45), containing 0.1% BSA. Then, PAds-derived cells were treated with increasing concentrations of GlaOC and GluOC (#4034491 and #4063516, respectively; BACHEM, Bubendorf, Switzerland) in presence of 1.5 mM extracellular calcium ([Ca^2+^]_o_) for 10 minutes for intracellular pathways’ investigation and for 6 hours for gene expression analysis. Tested GlaOC and GluOC concentrations, previously used by Pi et al. (18), were identified from preliminary dose-effect experiments in GPRC6A-HEK293A and CASR-HEK293A cells. Due to the limited number of cells obtained from each PAd sample, dose-effect curves could not be performed and the GlaOC and GluOC concentrations were restricted to 60 and 80 ng/mL. Untreated cells (NT) were considered as controls.

### RNA extraction and purification

Total RNA from PAds-derived cells cultures was isolated using TRIzol reagent (Invitrogen, ThermoFisher Scientific, Carlsbad, CA, USA) and genomic DNA contamination was removed by DNase I (Life Technologies, ThermoFisher Scientific, Carlsbad, CA, USA). Then, DNA-free RNA was quantified spectrophotometrically at Λ=260 nm.

### Real-time quantitative RT-PCR

Total cellular DNA-free RNA (300 ng) was reverse transcribed using the iScript cDNA Synthesis Kit (Bio-Rad, Hercules, CA, USA). Then, cDNA was amplified using TaqMan gene expression assay and a StepOnePlus™ Real-Time PCR System. The following probes were used: *PTH* Hs00757710_g1, *CASR* Hs01047795_m1, *CCND1* Hs00765553_m1, *GCM2* Hs00899403_m1, *VDR* Hs01045843_m1, *MEN1* Hs00365720_m1, *CDKNB1* Hs01597588_m1, and *TP73* Hs01056231_m1. The reference genes *HMBS* and *B2M* (Hs00609297_m1 and Hs99999907_m1, respectively) were used to normalize expression data and to obtain relative quantities using 2^(-ΔΔCt)^ formula. Where not otherwise specified, reagents and instruments were from Thermo Fisher Scientific (Carlsbad, CA, USA).

### Protein extraction and western blot analysis

Cells were homogenized using NP40 lysis buffer (FNN0021, ThermoFisher Scientific, Carlsbad, CA, USA) containing protease and phosphatase inhibitors to obtain total protein extracts. Membrane proteins were obtained from snap-frozen tissue sections (n=15) using a Dounce homogenizer and the Subcellular Protein Fractionation Kit for Tissues (ThermoFisher Scientific, Carlsbad, CA, USA). Protein concentration was measured using the Pierce BCA Protein Assay Kit (ThermoFisher Scientific, Carlsbad, CA, USA).

Twenty micrograms of total proteins per sample were loaded on a 10% or 7.5% SDS-PAGE, electrophoretically separated and then transferred to nitrocellulose membrane (Amersham Protran GE Healthcare Life Science, Chicago, Illinois, USA). For immunoblotting the following primary antibodies were used: GPRC6A (ab236964, Abcam, Cambridge, UK), CASR (ab19347, Abcam, Cambridge, UK), phosphorylated ERK and total ERK (#4370S and #9107S, respectively, Cell Signaling, Danvers, MA, USA), phosphorylated AKT and total AKT (#4060S and #4691S, respectively, Cell Signaling, Danvers, MA, USA), active unphosphorylated β-catenin (#8814, Cell Signaling, Danvers, MA, USA). Vinculin (ab129002, Abcam, Cambridge, UK) and GAPDH (ab9485, Abcam, Cambridge, UK) was used as loading controls for whole cell proteins, while Na^+^/K^+^-ATPase (ab76020, Abcam, Cambridge, UK) were used as loading control for membrane protein fractions. Binding of appropriate HRP-conjugated secondary antibodies was detected using the chemiluminescence ChemiDoc XRS System (Bio-Rad, Hercules, CA, USA). Analyses of bands densitometry were performed using Image Lab software (Bio-Rad, Hercules, CA, USA), and protein expression levels were normalized using vinculin, GAPDH, or Na^+^/K^+^-ATPase as reference.

ERK expression levels were considered as ratio of phosphorylated ERK/total ERK.

### Immunohistochemistry

Formalin-fixed paraffin-embedded (FFPE) samples were collected from 7 normal parathyroid glands (PaNs) incidentally removed from normocalcemic patients treated with thyroid surgery, and 12 PAds removed from PHPT patients. Parathyroid tissue sections were incubated with a rabbit monoclonal primary antibody specific for GPRC6A (TA208322, Origine, Rockville, MD, USA). Immunohistochemical staining was performed using the automatic staining BioGenex i6000 Automated Staining System (BioGenex, Fremont, CA, USA). Reactions were detected by Novolink Polymer Detection System (Novocastra Laboratories, Leica Microsystems, Newcastle, UK), according to the manufacturer’s instructions. Negative controls were incubated in the absence of primary antibody and human testis was used as positive control.

### Immunofluorescence

PAds-derived cells were fixed in 4% paraformaldehyde, permeabilized in 0.2% Triton X-100, and blocked in serum-free block protein solution (DAKO) for 1 h. Cells were incubated with primary antibodies, GPRC6A (TA308322, Origene, Rockville, MD, USA), PTH (sc-80924, Santa Cruz Biotechnology, Dallas, TX, USA), and GCM2 (sc-79491, Santa Cruz Biotechnology, Dallas, TX, USA), washed thrice in PBS, and then incubated with secondary antibodies conjugated with Alexa488 or Cy3 (1:100; Jackson Immuno Research, West Grove, PA, USA). Hoechst 33342 was used as nuclear stain (blue). As negative control, PBS was used instead of primary antibodies to exclude unspecific binding of secondary antibody. Images were obtained using fluorescence microscopy (Zeiss Axioskop 2 Plus, Zeiss OberKochen, Germany).

### CASR silencing in PAds-derived cells

PAds-derived cells were plated two days before transfection in antibiotic-free complete DMEM medium in 6-well plates. Cells were transiently transfected with 25 nM CaSR direct siRNA (L-005444-00-0005; ON-TARGET Plus siRNA SmartPool, Dharmacon, Lafayette, CO, USA) or 25 nM control siRNA (D-001810-10-05, ON-TARGETplus Non-targeting Pool, Dharmacon, Lafayette, CO, USA) in Opti-MEM medium (Gibco, ThermoFisher Scientific, Carlsbad, CA, USA), using DharmaFECT 1 (T-2001-02; Dharmacon, Lafayette, CO, USA) as transfection reagent. After 24 hours, transfected cells were treated for 10 minutes with either 80 ng/mL GlaOC or GluOC and analyzed by Western Blotting. All transfection conditions and reagent concentrations were previously optimized.

### GPCR6A and CASR transfections

HEK293A cells were seeded at a density of 1.2x10^5^ cells/well in 6-well plates and cultured in DMEM without penicillin-streptomycin. The following day, cells were transiently transfected with plasmid encoding for *GPRC6A* (sc-306340, Santa Cruz Biotechnology, Dallas, TX, USA), or with plasmid encoding for *CASR*, obtained by site-directed mutagenesis, as previously described (19). Two micrograms of GPRC6A plasmid were transfected using Lipofectamine3000 (L3000008, ThermoFisher Scientific, Carlsbad, CA, USA) in OptiMEM serum-free medium (GPRC6A-HEK293A cells). For CASR plasmid transfection, 4 micrograms of DNA were transfected using TurboFect Transfection Reagent (R0533, ThermoFisher, Carlsbad, CA, USA) in DMEM serum-free medium (CASR-HEK293A cells), following the manufacturer’s instructions. Preliminary experiments were performed to determine the optimal concentrations of both plasmids and to set up and optimize transfection conditions (**Supplementary Figures 1A, D**). GPRC6A and CASR transfections were functionally verified by documenting changes in pERK/ERK levels in response to stimulation with increasing concentrations of arginine and calcium as well as R568, respectively (**Supplementary Figures 1B, C, E–H**).

### Treatment of HEK293A cells with GlaOC and GluOC

Forty-eight hours after transfection, both GPRC6A-HEK293A and CASR-HEK293A cells were treated with increasing concentrations (20, 40, 60, 80 ng/mL) of GlaOC or GluOC, in the same experimental conditions used for PAds-derived cells. Cells were treated with the two isoforms of OC in presence of 1.5 mM [Ca^2+^]_o_ for 10 minutes for intracellular pathways investigation. Untreated cells (NT) were used as controls. The same experiments were also conducted on HEK293A cells transfected with the empty vector to verify the receptor specificity of the observed GlaOC or GluOC effects (data not shown).

### Apoptosis assays

For apoptosis experiments, PAds-derived cells and 8x10^4^ HEK293A cells transfected with GPRC6A or CASR plasmid (as described above) were seeded in 96-well plates. Cleaved Caspase 3/7 activity levels were measured using the Apo-One^®^ Homogeneous Caspase 3/7 assay (Promega Corp., WI, USA) that provides a profluorescent substrate and a cell lysis/activity buffer for Caspase 3/7 (DEVDase) activity assay. After induction of apoptosis with 2 μM staurosporine (Merk Millipore, Burlington, MA, USA) and treatment with GlaOC and GluOC for 5 hours, 100 μl of Apo-One was added to each well, incubated for 2-18 hours and then fluorescence levels were measured (485Ex/527Em) according to the manufacturer’s instructions.

### Statistics

Densitometric data were log2 transformed, analyzed by One-way ANOVA adjusted for multiple comparisons and presented as mean±SEM. All data related to protein expression were normalized on the levels detected in untreated conditions.

Gene expression data were log2 transformed and presented as mean±SEM. Differences among the levels at each time point experiment were tested by One-way ANOVA analysis adjusted for multiple comparisons. Correlations between gene expression and biochemical parameters were analyzed by Pearson coefficient of correlation.

A probability value (P) less than 0.05 was considered statistically significant. Statistical analyses were performed using Prism v6.0 (GraphPad Inc., San Diego, CA, USA).

## Results

### Effects of GlaOC and GluOC stimulations on intracellular signaling pathways in human parathyroid adenomatous cells

We first tested the hypothesis that GlaOC or GluOC or both can affect parathyroid tumor cell biology. The OC putative receptor GPRC6A (20) is coupled to the modulation of the extracellular signal-regulated kinases (21–23), of the protein kinase B (24, 25), and of the WNT/β-catenin signaling (26). Therefore, we investigated the effect of both GlaOC and GluOC on these intracellular signaling pathways in parathyroid adenomas (PAd)-derived cells. Specifically, changes in the levels of phosphorylated and total extracellular signal-regulated kinases (pERK/ERK), of phosphorylated and total protein kinase B (pAKT/AKT), and of the active unphosphorylated β-catenin in whole cell protein extracts were quantified by western blot analysis. PAds-derived primary cell preparations were incubated with increasing concentrations of GlaOC (60-80 ng/mL) and GluOC (60-80 ng/mL) for 10 minutes.

**Figure 1:**
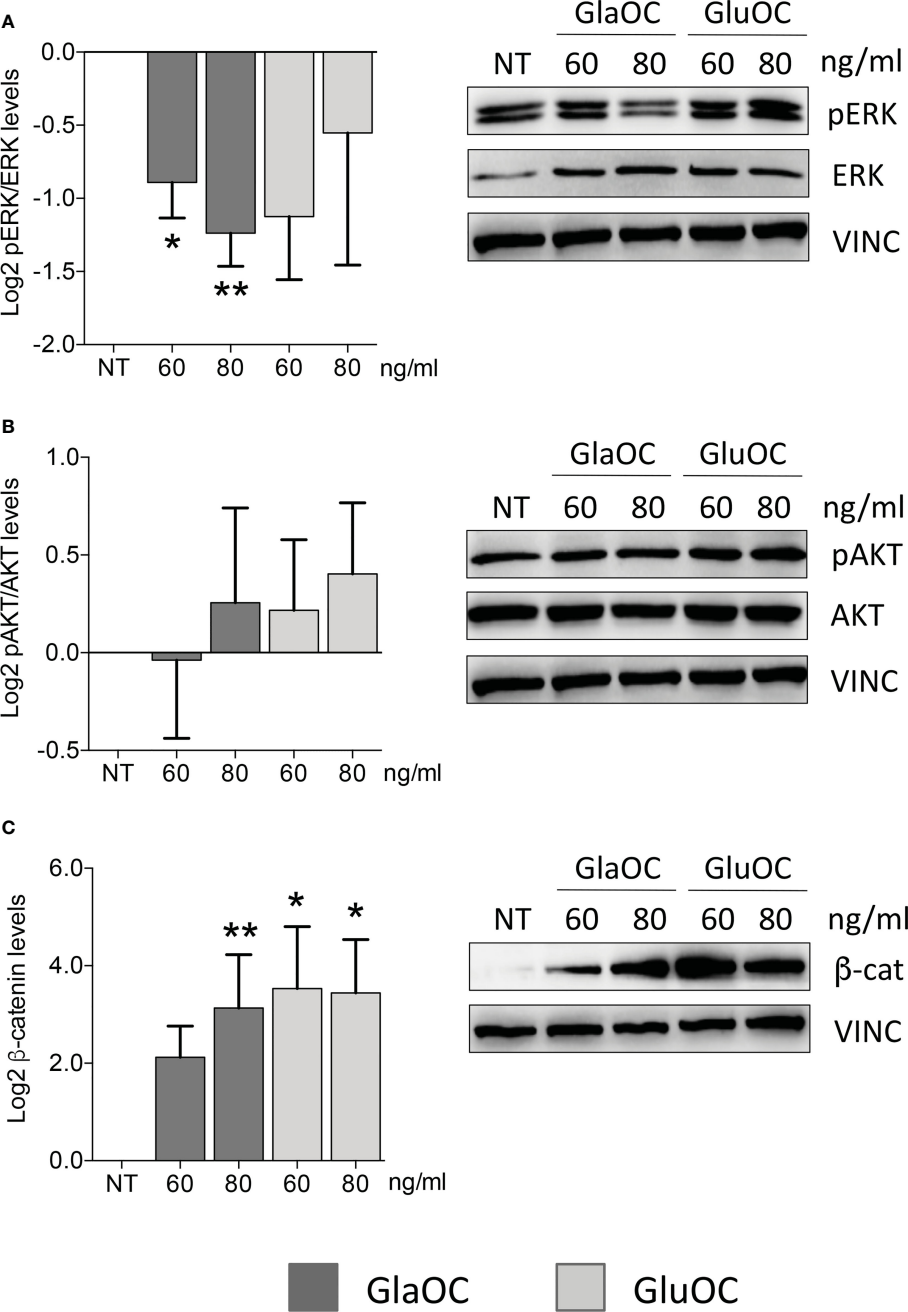
Effects of the GlaOC and GluOC stimulation on intracellular signaling pathways in human parathyroid cells. PAds-derived cells were incubated for 10 minutes with increasing concentrations of GlaOC (dark grey columns; 60-80 ng/mL) and GluOC (light grey columns; 60-80 ng/mL) (n=4). A representative western blot is shown for each experimental condition and data are expressed as mean ± SEM. **(A)** GlaOC and GluOC effects on basal pERK/ERK levels (*, P=0.019; **, P=0.004). **(B)** GlaOC and GluOC effects on basal pAKT/AKT levels. **(C)** GlaOC and GluOC effects on basal active β-catenin levels (*, P=0.048; **, P=0.042). VINC, vinculin was used as loading control.

GlaOC, at both concentrations, inhibited the basal pERK/ERK levels of about two folds, while GluOC-induced changes were not significantly different from basal conditions (n=4) (**Figure 1A**). The two isoforms of OC did not exert any significant effect on pAKT/AKT levels (**Figure 1B**), while both GlaOC and GluOC stimulated the basal active β-catenin levels of about 8 folds (n=4) (**Figure 1C**).

### Effects of GlaOC and GluOC stimulations on the expression of specific genes in human PAds-derived cells

**Figure 2:**
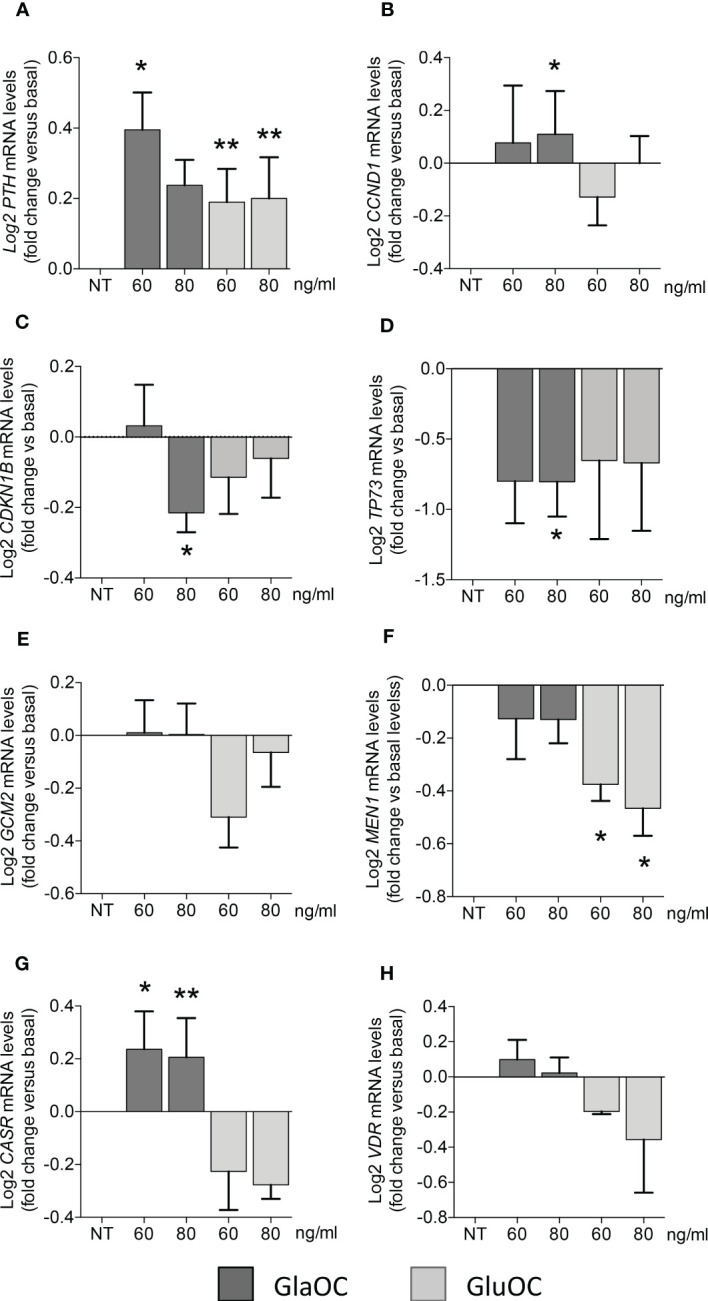
Effects of GlaOC and GluOC stimulation on the expression of specific genes in PAds-derived cells. PAds-derived cells were incubated for 6 hours with increasing concentrations (60-80 ng/mL) of GlaOC (dark grey columns) and GluOC (light grey columns). Data were expressed as fold change versus levels in non treated conditions (NT) and presented as mean ± SEM. GlaOC and GluOC modulated the expression of **(A)** *PTH* mRNA levels (*, P=0.015; **, P=0.008), **(B)** *CCND1* transcripts (*, P=0.002), **(C)** *CDKN1B* mRNA levels (*, P=0.041), and **(D)** *TP73* mRNA levels (*, P=0.042). Effects of GlaOC and GluOC on parathyroid specific transcription factors **(E)** *GCM2* mRNA levels, **(F)** *MEN1* mRNA levels (*, P=0.0001), and on **(G)** the receptors *CASR* (*, P=0.002; **, P=0.014) and **(H)** VDR mRNA levels are shown.

Incubation for 6 hours of PAds-derived cell preparations (n=8) with increasing concentrations (60 and 80 ng/mL) of GlaOC elicited significant increases of the expression levels of the parathyroid specific gene *PTH* (**Figure 2A**). Modest but significant increases of *CCND1* transcripts were elicited by 80 ng/mL GlaOC (**Figure 2B**), in association with decreases in *CDKN1B* and *TP73* mRNA levels (**Figures 2C, D**, respectively). Besides, GlaOC did not affect the expression levels of the transcription factors *GCM2* (**Figure 2E**) and *MEN1* (**Figure 2F**). GlaOC stimulated the expression of *CASR* (**Figure 2G**), but not that of *VDR* mRNA levels (**Figure 2H**). As far as GluOC was concerned, increasing concentrations (60 and 80 ng/mL) induced *PTH* expression levels (**Figure 2A**), while GluOC did not affect the expression of *CCND1* (**Figure 2B**), *CDKN1B* (**Figure 2C**), *TP73* (**Figure 2D**), *GCM2* (**Figure 2E**), *CASR* (**Figure 2G**) and *VDR* (**Figure 2H**). At variance, GluOC reduced *MEN1* mRNA levels (**Figure 2F**).

### GPCR6A expression in human parathyroid gland

**Figure 3:**
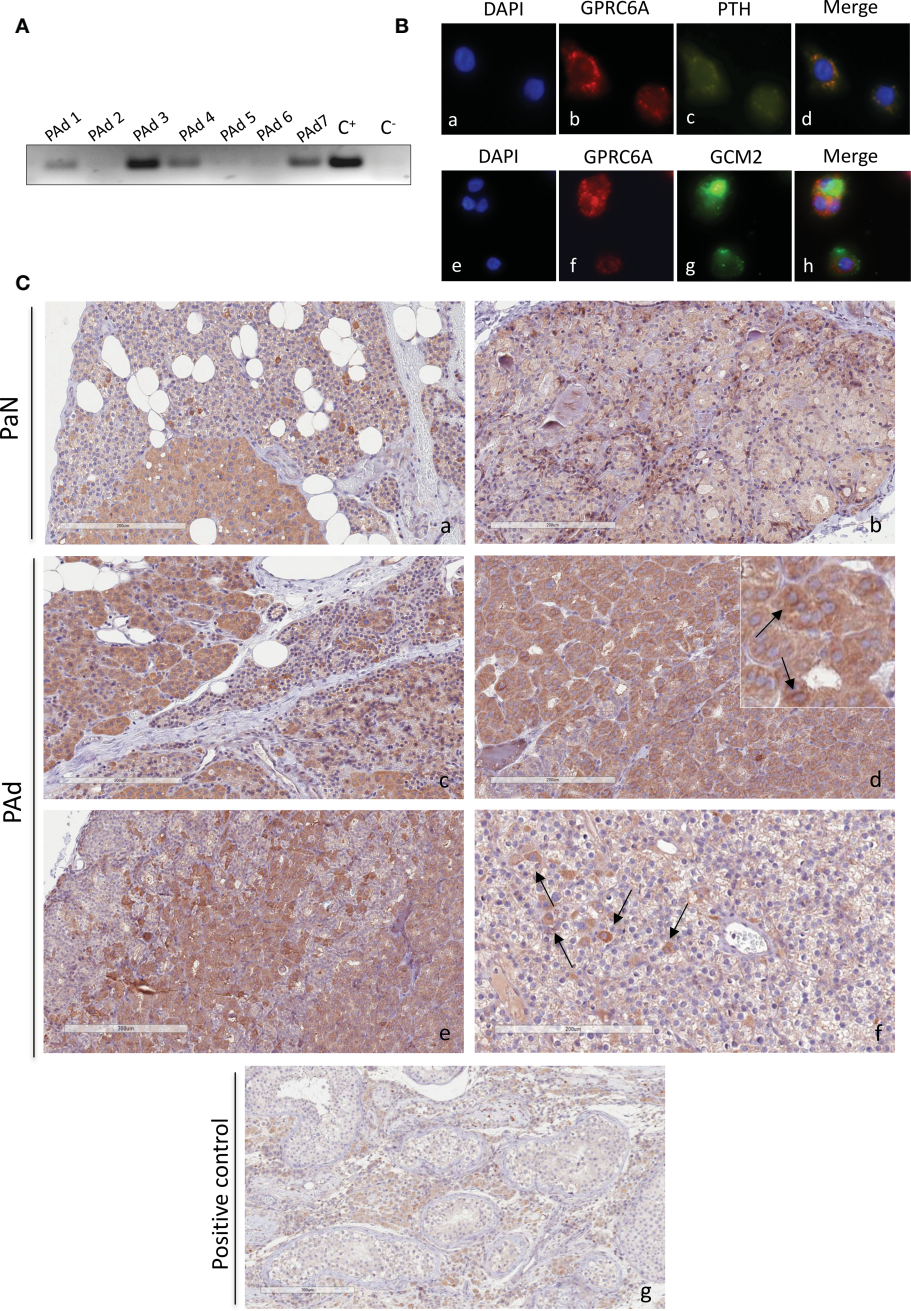
GPCR6A expression in human parathyroid cells. **(A)** *GPRC6A* transcripts were variably detected by RT-PCR in total RNA from PAds (n=7) of PHPT patients; C^+^, plasmid encoding GPRC6A; C^-^, water. **(B)** Immunofluorescence of short-term cultured PAds-derived cells showed cytoplasmic and membrane expression of GPRC6A (red, **b, f**); PTH (green, **c**) co-expressed with GPRC6A (merge, **d**) GCM2 (green, **g**) co-expressed with GPRC6A (merge, **h**). **(C)** Immunohistochemistry by a specific anti-GPRC6A antibody in normal parathyroid glands from normocalcemic patients with thyroid diseases **(a, b)** and in parathyroid adenomas (panels **c–f**). **(g)** Human testis with GPRC6A-expressing Leydig cells were used as positive control. Insert in **(d)** shows cells with GPRC6A expression at membrane level. Magnification 20X; bars, 200 μm.

Our data suggests that GlaOC and GluOC induce biologic responses in PAds-derived cells, though at different extents, in term of modulation of intracellular signaling pathways and of parathyroid specific genes expression. These patterns of responses may be mediated by the membrane receptor GPRC6A. Therefore, we investigated GPRC6A expression in human parathyroid glands. Human *GPRC6A* transcripts were detected in total RNA extracted from a series of PAds derived from patients affected with sporadic PHPT (**Figure 3A**). Immunofluorescence with specific antibodies demonstrated that the GPRC6A protein was expressed in the PTH-expressing and GCM2-expressing PAds-derived cells (**Figure 3B**). GPRC6A expression was also investigated in normal parathyroid glands derived from normocalcemic subjects due to accidentally excision during thyroid surgery. IHC detected GPRC6A both at membrane and cytoplasmic levels, showing heterogeneity within sections from normal parathyroid glands (**Figure 3C, a, b**), where GPRC6A-expressing cells were scattered throughout the parenchyma or clusterized in defined areas. A similar pattern of expression was detected in sections from PAds (**Figure 3C, c-f**). Of note, GPRC6A protein was more abundant in parathyroid samples than in the human testicular Leydig cells (**Figure 3C, g**), used as positive control.

### Relationships among GPRC6A, CASR expression levels and clinical features of parathyroid tumors

**Figure 4:**
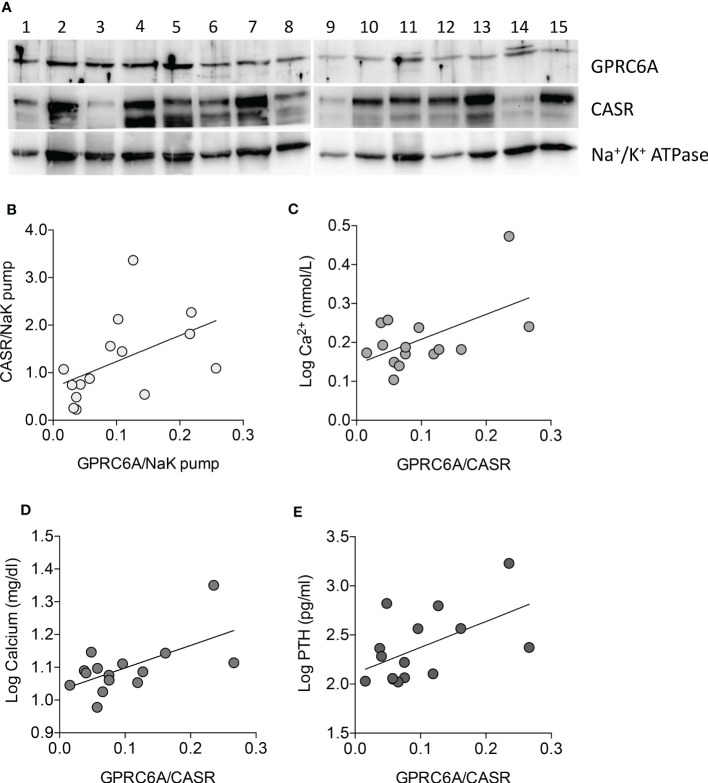
GPRC6A and CASR expression in membrane protein fractions from PAds and correlation with clinical features. **(A)** Western blot analysis of GPRC6A and CASR expression in membrane protein fractions from a series of PAds (n=15); GPRC6A specific band was detected at 105 kDa; specific CASR bands were detected at 130 and 150 kDa; Na^+^/K^+^ ATPAse was used as loading control. **(B)** Correlation between GPRC6A and CASR membrane proteins in the PAds series (r=0.618, P=0.014 by Spearman coefficient of correlation). **(C)** Correlation between GPRC6A/CASR ratio and plasma ionized calcium levels, expressed as log2 (r=0.552, P=0.033 by Pearson coefficient of correlation). **(D)** Correlation between GPRC6A/CASR ratio and serum total calcium levels, expressed as log2 (r=0.602, P=0.018 by Pearson coefficient of correlation). **(E)** Correlation between GPRC6A/CASR ratio and plasma PTH levels, expressed as log2 (r=0.539, P=0.038 by Pearson coefficient of correlation).

GPRC6A and CASR belong to class C G-protein coupled receptors and share a high degree of homology (27). The expression levels of GPRC6A and CASR proteins were analyzed by western blot in membrane protein fractions from 15 PAds (**Figure 4A** and **Supplementary Figure 2A**). Patients’ clinical and biochemical features are reported in **Supplementary Table 1**. Both receptors were variably expressed among PAds, though their expression levels were positively correlated (r=0.618, P=0.014) (**Figure 4B**). Of note, considering the ratio between GPRC6A and CASR expression levels, PAds expressing higher levels of GPRC6A were associated with higher levels of circulating ionized calcium (r=0.552, P=0.033)(**Figure 4C**), total calcium (r=0.602, P=0.018)(**Figure 4D**), and PTH (r=0.539, P=0.038)(**Figure 4E**).

### Effects of GPRC6A activation by GlaOC and GluOC in HEK293A cells transfected with GPRC6A

To define the contribution of GPRC6A in the signaling response to GlaOC and GluOC stimulation in PAds, we studied pERK/ERK and β-catenin OC-stimulated responses using HEK293A cells transfected with the *GPRC6A* gene (GPRC6A-HEK293A cells) as experimental model (**Supplementary Figures 1A–C**). PhosphoAKT/AKT signaling was not evaluated as in PAds-derived cells it did not show significant changes in response to stimulation with isoforms of OC. Experiments were repeated four times in presence of 1.5 mM extracellular calcium ([Ca^2+^]_o_).

**Figure 5:**
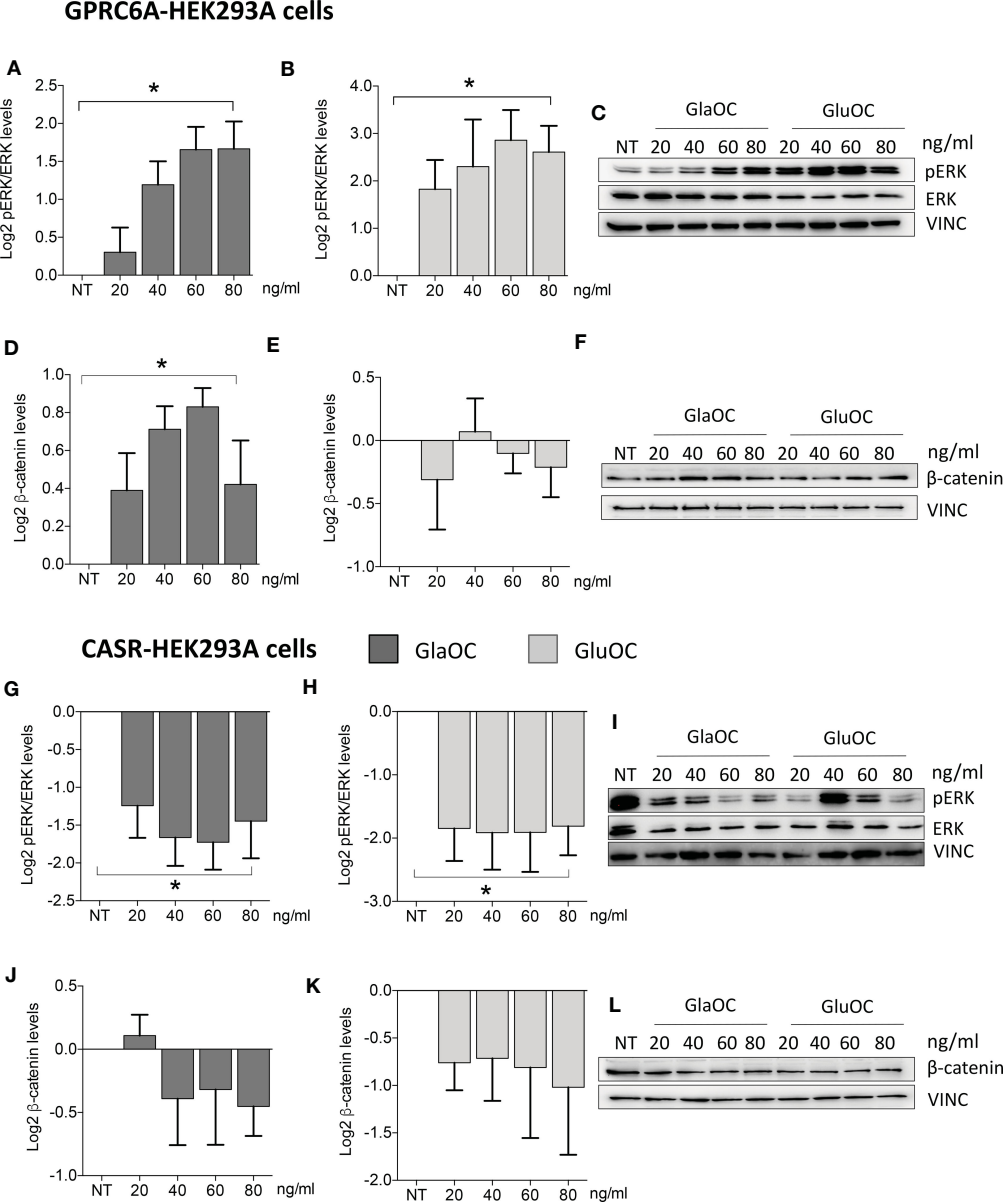
Effects of the treatment with GlaOC or GluOC in HEK293A cells transfected with GPCR6A and with CASR. HEK293A cells transiently transfected with GPRC6A (GPRC6A-HEK293A) or CASR (CASR-HEK293A) were incubated for 10 minutes with increasing concentrations (20, 40, 60, 80 ng/mL) of GlaOC (dark grey columns) and GluOC (light grey columns). Data were log2 transformed and presented as mean±SEM. **(A, B)** Effects of increasing concentrations of GlaOC and GluOC on pERK/ERK levels (*, P=0.006 and P=0.05, respectively) in GPRC6A-HEK293A cells. **(C)** Representative western blot. **(D, E)** Effects of increasing concentrations of GlaOC and GluOC on active β-catenin levels (*, P=0.025) in GPRC6A-HEK293A cells and representative western blot **(F)**. **(G, H)** Effects of increasing concentrations of GlaOC and GluOC on pERK/ERK levels (*, P=0.023 and P=0.048, respectively) in CASR-HEK293A cells and representative western blot **(I)**. (**J, K)** Effects of increasing concentrations of GlaOC and GluOC on β-catenin levels in CASR-HEK293A cells and representative western blot **(L)**.

Increasing concentrations of both GlaOC and GluOC significantly stimulated of about 4 folds basal pERK/ERK levels (**Figures 5A–C**). Of note, this pattern of response was opposite to that observed in PAds-derived cells. Increasing concentrations of GlaOC induced small but significant increases of the basal active β-catenin levels (**Figure 5D, F**), while GluOC did not affect them (**Figure 5E, F**).

### Effects of CASR activation by GlaOC and GluOC in HEK293A cells transfected with CASR

HEK293A cells transfected with CASR (CASR-HEK293A cells) (**Supplementary Figures 1D-H**) were used to test the hypothesis that GlaOC and/or GluOC can activate CASR and to investigate the effect of the GlaOC/GluOC-CASR activation on pERK/ERK and β-catenin signaling (n=4). In presence of 1.5 mM [Ca^2+^]_o_, increasing concentrations of both GlaOC and GluOC significantly inhibited basal pERK/ERK levels (**Figures 5G–I**), resembling the inhibitory effect observed in PAds-derived cells and suggesting that it is likely mediated by OC-stimulated CASR. Unlike what happened in PAds-derived cells, both GlaOC and GluOC did not exert any significant change in the basal active β-catenin levels in CASR-HEK293 cells (**Figures 5J–L**).

### Effects of CASR silencing on GlaOC and GluOC stimulated intracellular signaling pathways in PAds-derived cells

**Figure 6:**
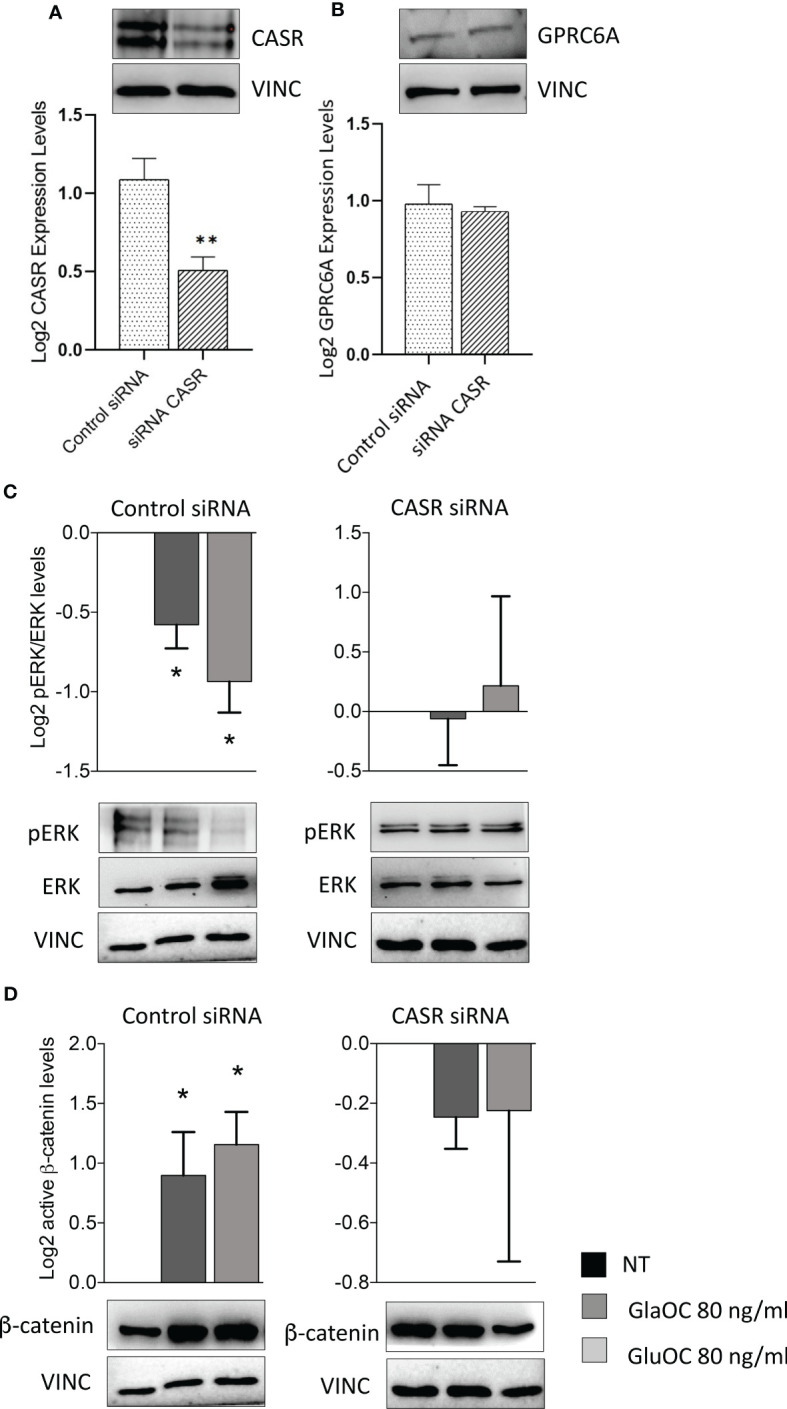
Effect of CASR silencing on the GlaOC/GluOC-activated signaling in PAds-derived cells. **(A)** Effect of the *CASR* silencing on CASR protein expression (**, P=0.0043). **(B)** Effect of the *CASR* silencing on GPRC6A protein levels; expression levels are presented as fold changes versus untreated conditions incubated with control siRNA, and log2 transformed. **(C)** Effects of GlaOC (dark grey columns) and GluOC (light grey columns) stimulation in PAds-derived cell preparations treated with control siRNA and with CASR siRNA on pERK/ERK levels (*, P<0.05). **(D)** Effects of GlaOC (dark grey columns) and GluOC (light grey columns) stimulation in PAds-derived cell preparations treated with control siRNA and with CASR siRNA on active β-catenin levels (*, P<0.05). Densitometric data were log2 transformed and presented as mean±SEM. A representative western blot is shown for each experimental condition.

To define the specific role of OC-activated CASR, experiments modulating intracellular signaling pathways were repeated in PAds-derived cell preparations (n=6) with transiently silenced *CASR*. CASR expression resulted to be reduced of at least 50% in the total protein extracts from all cell preparations (**Figure 6A**), while GPRC6A protein expression levels were unaffected by CASR silencing (**Figure 6B**). Immunoblots of GPRC6A were faint in 2 out 6 primary cell cultures, in line with the highly variable expression observed in IHC sections, therefore, the cell cultures were excluded from the analysis. In PAds expressing GPRC6A and CASR, reduction of CASR expression, blunted the inhibitions of pERK/ERK levels induced by GlaOC and GluOC (80 ng/mL)(**Figure 6C**). At variance, GlaOC and GluOC-induced increases in active β-catenin levels were unaffected by CASR silencing (**Figure 6D**), suggesting that β-catenin may be mainly modulated by GPRC6A activation.

### Effects of GlaOC and GluOC stimulation on apoptosis in PAds-derived cells, GPRC6A-HEK293A, and CASR-HEK293A cells

**Figure 7:**
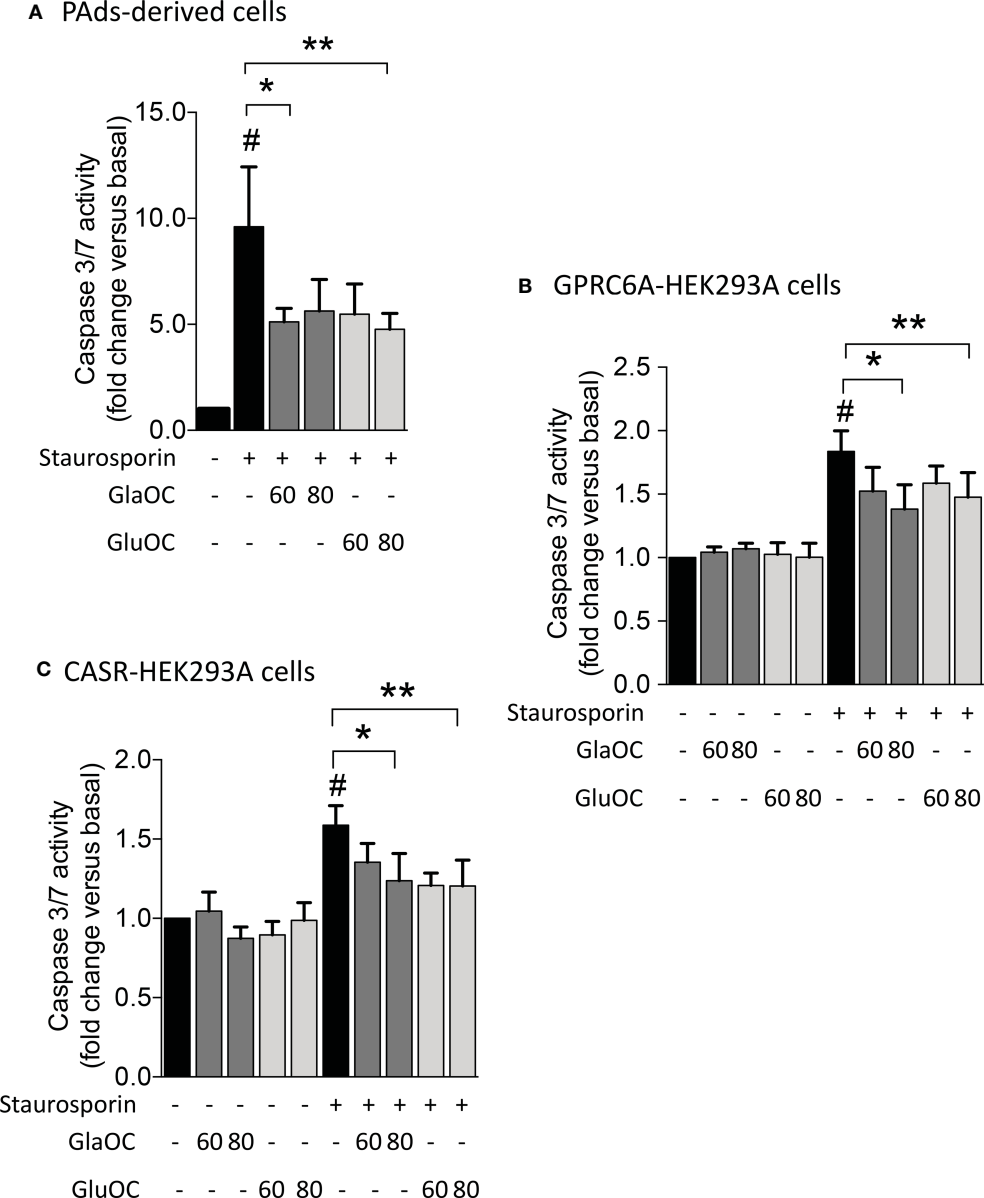
Effects of GlaOC and GluOC stimulation on staurosporin-induced apoptosis. **(A)** Effects of 60-80 ng/mL GlaOC (dark grey columns) and GluOC (light grey columns) on staurosporin-induced apoptosis in PAds-derived cells (#, P=0.022 versus basal levels; *, P=0.050 vs staurosporin treated cells; **, P=0.045 vs staurosporin treated cells). **(B)** Effects of 60-80 ng/mL GlaOC and GluOC on staurosporin-induced apoptosis in GPRC6A-HEK293A cells (#, P=0.036 vs basal levels; *, P=0.013 vs staurosporin treated cells; **, P=0.087 vs staurosporin treated cells). **(C)** Effects of 60-80 ng/mL GlaOC and GluOC on staurosporin-induced apoptosis in CASR-HEK293A cells (#, P=0.042 vs basal levels; *, P=0.010 vs staurosporin treated cells; **, P=0.040 versus staurosporin treated cells).

Finally, considering the inhibitory effect of GlaOC and GluOC in PAds-derived cells on the expression of *TP73*, which is a master of apoptotic pathways (28), we tested the effect of GlaOC and GluOC on staurosporin-induced caspase 3/7 activity, known to have executioner roles for apoptosis (29). In PAds-derived cells (n=4), both GlaOC and GluOC significantly reduced the staurosporin-induced caspase 3/7 activities of about 40% of the basal levels, after 5 hours incubation (**Figure 7A**). Though HEK293A cells were less sensitive to the staurosporin apoptotic stimulus after 5 hours than PAds-derived cells, in GPRC6A-HEK293A cells (n=3) (**Figure 7B**) as well as in CASR-HEK293A cells (n=3) (**Figure 7C**), staurosporin-induced caspase 3/7 activities were reduced of about 25% by treatments with 80 ng/mL of GlaOC and GluOC, similarly to the effect detected in PAds-derived cells. These observations suggest that the OC antiapoptotic effect may be mediated by both GPRC6A and CASR.

## Discussion

The present study firstly provides evidence suggesting that the bone-derived OC may modulate parathyroid tumor cell function in PHPT-related parathyroid adenomas. Osteocalcin, which is variably γ-carboxylated to three glutamic residues (3) and is released by osteoblasts, recently received researchers’ attention, who defined its hormonal function. Besides the role in bone matrix mineralization (30), OC modulates, through the activation of the class C G-protein coupled receptor GPRC6A, the function of β cells of pancreatic islets, skeletal muscle fibers, adipose tissue, brain, and testes (31). In different cell models, OC modulates intracellular ERK, AKT, and β-catenin pathways through activation of GPRC6A (18, 25, 26).

Previous studies described the important role of the MAPK/ERK pathway in parathyroid cell function, and its dysregulation in parathyroid adenomas, where *HRAS*, *ARAF*, and *MEK1* genes are up-regulated (32) and ERK is hyperactivated (19, 33, 34). For these reasons, we investigated the effect of OC modulation on ERK pathway in PAds-derived cells. We demonstrated that GlaOC and GluOC inhibited basal pERK/ERK levels. Of note, OC exerts an opposite effect with respect to the stimulation induced by [Ca^2+^]_o_-activated CASR on intracellular pERK/ERK levels (19, 33, 34).

Though AKT and β-catenin signaling has been poorly investigated in parathyroid tumor cell biology so far, the PI3K/AKT/mTOR pathway was impaired in about one fifth of the parathyroid cancers (35, 36), suggesting a role of the AKT pathway in parathyroid cell pathophysiology. Similarly, data about the nuclear β-catenin accumulation in parathyroid tumors are controversial; nonetheless, the WNT/β-catenin pathway can play a role in parathyroid cell biology as its activation by lithium chloride inhibited the expression of the embryonic transcription factor TBX1 (37) and increased the aberrant expression of miR-372 in PAds-derived cells (38). Present data suggest that pAKT/AKT pathway is poorly modulated by OC in PAds-derived cells, while GlaOC and GluOC significantly increased active β-catenin levels.

Furthermore, the two OC isoforms not only modulate intracellular signaling, but also affect the expression of parathyroid specific genes. PAds-derived cells responded to both GlaOC and GluOC stimulations by increasing the expression levels of the parathyroid specific gene *PTH*. The observed increase of *PTH* gene expression is consistent with the inhibitory effect of GlaOC and GluOC on pERK/ERK levels. It has been demonstrated that abrogation of the ERK pathway abolishes the inhibitory effect of 1.5 mM [Ca^2+^]_o_ on PTH release from normal parathyroid cells supporting the role of MAPK in the modulation of the PTH secretion (39). Similarly, inhibition of the ERK pathway by FGF23 stimulates the release of PTH in rats with secondary hyperparathyroidism (40). Considering that PTH itself stimulates the release of OC by osteoblasts (4), the detected PTH stimulation by both GlaOC and GluOC suggests the existence of a self-regulatory positive feedback loop between PTH and OC.

GlaOC, but not GluOC, also increased the expression of the genes *CCND1* and *CASR*, and decreased the expression of *CDKN1B* and *TP73*, showing a heterogeneous response to GlaOC and GluOC in terms of PAds gene modulation. Of note, CCND1 has been previously shown to be stimulated by CASR activation (41). In line with this view, GluOC, but not GlaOC, inhibited the expression of the tumor suppressor gene *MEN1*. The effects observed on *CCND1* and *MEN1* suggested a possible modulation of the parathyroid tumor cell proliferation.

Admittedly, the modulation of the parathyroid genes’ expression is of modest entity: it may be related to the extremely variable expression of both CASR and GPRC6A detected in the different PAds.

IHC revealed GPRC6A specific staining in both normal and adenomatous parathyroid glands with intensity even more consistent compared with that detected in human testicular Leydig cells (42). GPRC6A was expressed by the endocrine parathyroid cells co-expressing PTH and the specific parathyroid transcription factor GCM2. Furthermore, the GPRC6A expression was heterogeneous in the single parathyroid tumor: intensively positive cells were scattered through cells with weak membrane positive staining. Similarly, CASR, which is highly homolog with GPRC6A, was heterogeneously expressed in PAds, in agreement with previous reports (39). Of note, the expression levels of the two receptors showed a positive correlation and were positively correlated with the circulating levels of ionized calcium and PTH in PHPT patients, suggesting that also GPRC6A may be involved in modulating PTH secretion and in determining the clinical presentation of PHPT. Therefore, the effects determined by GlaOC and GluOC through the activation of GPRC6A or CASR, on pERK/ERK and β-catenin signaling, were dissected using HEK293A cells transiently transfected with the human GPRC6A or with the human CASR, as experimental models.

In GPRC6A-HEK293A cells, GlaOC and GluOC increased basal pERK/ERK levels exerting opposite effects with respect to those detected in PAds-derived cells. GPRC6A is mainly coupled to G protein q/11 in HEK293 cells (43), and our data may suggest Gq/11 coupling mediating GlaOC and GluOC-stimulated ERK signalling.

Interestingly, in CASR-HEK293A cells, GlaOC and GluOC inhibited basal pERK/ERK levels, suggesting that GlaOC and GluOC in PAds likely act through the activation of CASR. In line with this observation, the reduction of CASR expression in PAds-derived cells blunted the pERK inhibition induced by GlaOC and GluOC, confirming that in parathyroid tumor cells pERK/ERK modulation by GlaOC and GluOC is mainly mediated by CASR activation.

Resembling what detected in PAds-derived cells, in GPRC6A-HEK293A cells GlaOC increased the basal levels of active β-catenin. In silenced PAds-derived cells, the reduction of CASR expression abolished the increases in active β-catenin levels, suggesting that also β-catenin is mainly modulated by GlaOC- and GluOC-activated CASR.

Finally, GlaOC and GluOC had protective effects on induction of apoptosis in PAds-derived cells as well as in GPRC6A-HEK293A and CASR-HEK293A cells. In contrast with what previously described in other studies (44, 45), not only GluOC but also GlaOC partially prevented apoptosis acting through GPRC6A and CASR.

Interestingly, at variance with data reported in mouse models, where GluOC showed endocrine function (46), our data obtained both in human primary tumor cell and in human cell line cultures, indicated GlaOC as the bioactive molecule able to more consistently elicit modulation of intracellular signaling pathways and of gene expression. Differences observed between mice, where the hormone function is evident for GluOC, and humans, where GlaOC may act as modulator of the parathyroid function in adenomatous glands and of the skeletal muscle (47), may be related to the different carboxylation status: OC is γ-carboxylated on glutamic acids (Glu) 13, 17 and 20 of protein in mouse, and on Glu 17, 21 and 24 in humans (48).

Finally, according to previously published studies, average circulating levels of total OC range between 9 and 42 ng/mL, in adult women (https://www.mayocliniclabs.com/test-catalog/overview/80579#Clinical-and-Interpretive). To our knowledge, no data are available from PHPT patients. We tested in the *in vitro* experiments OC concentrations sensibly higher, i.e., supraphysiological, than the circulating ones in humans. This might appear as a simplification, or a limitation of the study; however, it should be considered that PAds-derived cells rapidly develop reduced sensitivity to [Ca^2+^]_o_ due to CASR downregulation implying the need to stimulate cells with supraphysiological [Ca^2+^]_o_ (2.5-5.0 mM) (39) and, since the hypothesized CASR-mediated effects of OCs, the use of supraphysiological doses was acknowledged. Moreover, the OCs concentrations used are in line with doses already reported in other *in vitro* models derived from different species (18, 21).

**Figure 8:**
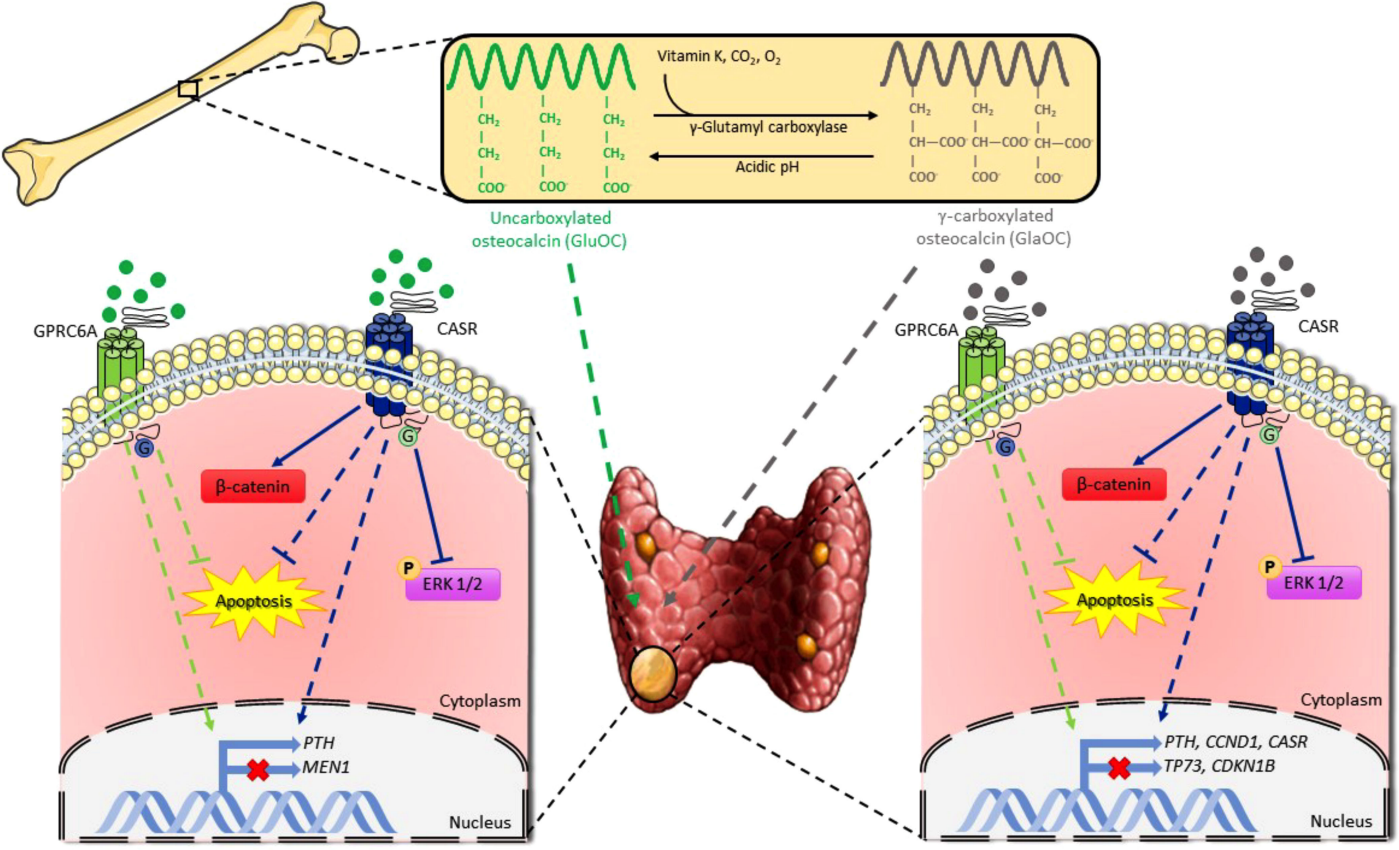
Schematic representation of osteocalcin effects on parathyroid tumor cell signaling pathways. GlaOC and GluOC, activating GPRC6A and CASR, modulate parathyroid gene expression and partially prevent apoptosis. GlaOC also reduces ERK phosphorylation and increases β-catenin mainly acting through CASR. Part of this figure was created using images from Servier Medical Art, licensed under a Creative Commons Attribution 3.0 Unported License (https://smart.servier.com).

In conclusion, GlaOC and GluOC released from bone matrix may affect parathyroid tumor cell function, apoptosis, and indirectly proliferation (**Figure 8**). They may contribute to defining the heterogeneity of the biochemical phenotype associated with parathyroid adenomas in PHPT patients, promoting development of autonomous PTH secretion. It is tempting to speculate that persistent high bone turnover with release of GlaOC and GluOC, as occurs in postmenopausal bone demineralization, may desensitize parathyroid cells towards [Ca^2+^]_o_, and it may also promote parathyroid cell proliferation, and protect parathyroid cells from apoptotic stimuli, ultimately contributing to parathyroid hyperplasia. Parathyroid hyperplasia associated with high circulating GlaOC and GluOC concentrations might benefit of antiresorptive agents as inhibition of the bone turnover and consequent decrease in GlaOC and GluOC release may improve parathyroid cells sensitivity to [Ca^2+^]_o_. However, further studies aimed to elucidate the role of GlaOC and GluOC in modulating the mineral metabolism and the clinical effects should be conceived.

## Data availability statement

The datasets presented in this study can be found in online repositories. The names of the repository/repositories and accession number(s) can be found below: https://doi.org/10.5281/zenodo.5069796.

## Ethics statement

The studies involving human participants were reviewed and approved by Ospedale San Raffaele Ethical Committee, protocol no. GPRC6A PARA, 07/03/2019; CE40/2019. The patients/participants provided their written informed consent to participate in this study.

## Author contributions

CV and GT contributed equally to this work. Conceptualization, CV and SC; methodology, CV, GT, IF, validation, CV, GT; formal analysis, CV, GT; investigation, CV,GT, IF, VV, RM, LV, PD, FP; resources GL, SC; data curation, SC; writing – original draft preparation, SC; writing – review and editing, CV, GT and SC; supervision, SC; project administration, SC; funding acquisition, SC. All authors contributed to the article and approved the submitted version.

## Supplementary material

Supplementary Figure 1 Functional characterization of the GPRC6A-HEK293A cells and CASR-HEK293A cells. **(A)** Western blot detection of GPRC6A protein in GPRC6A-HEK293A cells as a band of 105 kDa. **(B)** Increases of the basal pERK/ERK levels after stimulation with 20 and 30 mM L-arginine of GPRC6A-HEK293A cells transiently transfected with different amount of GPRC6A plasmid (2 and 4 μg). **(C)** Densitometric analysis of the western blot shown in b. **(D)** Western blot detection of CASR protein in CASR-HEK293A cells as two bands of 100 and 150 kDa. **(E)** Significant increases of the basal pERK/ERK levels after stimulation of CASR-HEK293A cells with increasing concentrations of [Ca^2+^]_o_ (1.0, 3.0 and 5.0 mM). **(F)** Densitometric analysis of the western blot shown in e. **(G)** Basal pERK/ERK level increases after stimulation with different concentrations (50 and 100 nM, 0.5 and 5.0 μM) of the potent CASR agonist R568 (Cayman Chemical Company; Ann Arbor, Michigan, USA). **(H)** Densitometric analysis of the western blot shown in g.

Supplementary Figure 2
** (A)** Representative western blot analysis of the GPRC6A and CASR proteins’ expression in membrane protein fractions from 2 PAds (TBP ab51841, Abcam, Cambridge, UK). **(B)** Exemplificative western blots obtained in a GPRC6A-expressing PAd transfected with Control siRNA and with CASR siRNA.

Supplementary Table 1 Clinical and biochemical features of PHPT patients, whose surgically removed PAds were analyzed for CASR and GPRC6A membrane expression. Patient ID, identification corresponding to that used in; age, PHPT patient age at diagnosis; BMI, body mass index; Ca^2+^, plasma ionized calcium; SCa, serum total calcium; PTH, plasma parathormone; creat, serum creatinine; tumor size, maximal diameter of the surgically removed parathyroid adenoma; kidney, diagnosis of kidney stones symptomatic disease and asymptomatic stones detected by imaging); bone, diagnosis of osteopenia/osteoporosis according WHO criteria (49).
