# Supplementary figures and images for: Osteocalcin modulates parathyroid cell function in human parathyroid tumors
Source: Front Endocrinol (Lausanne). 2023 Mar 30;14:1129930. doi: 10.3389/fendo.2023.1129930 (PMC10098338; doi:10.3389/fendo.2023.1129930)

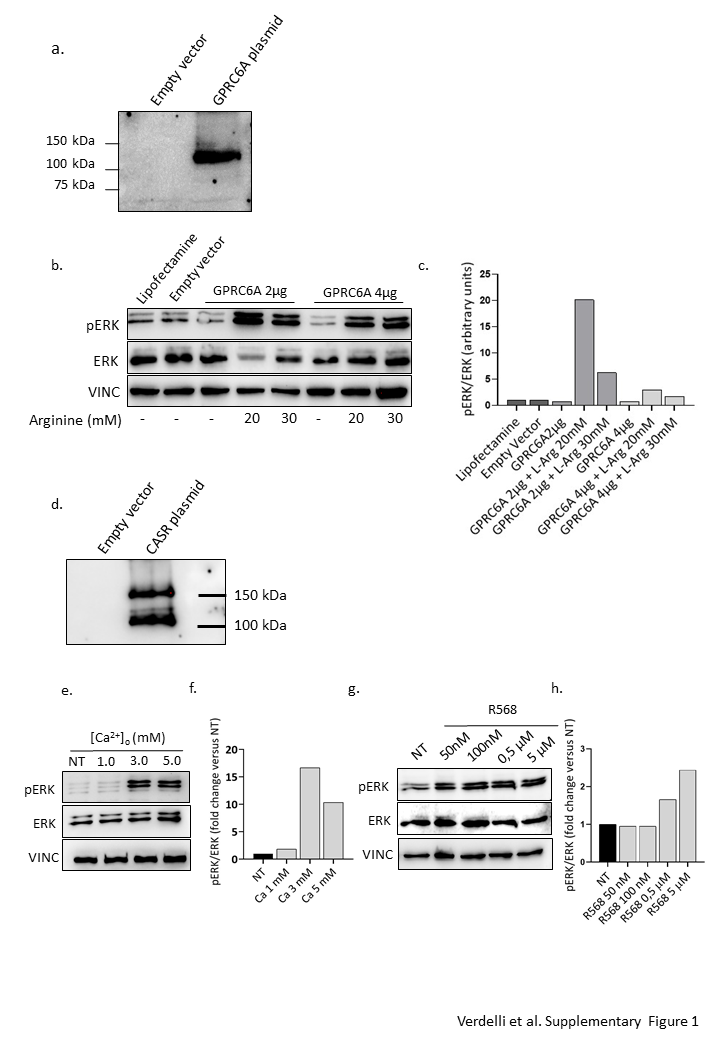

Supplement: Supplementary Figure 1 — Functional characterization of the GPRC6A-HEK293A cells and CASR-HEK293A cells. (A) Western blot detection of GPRC6A protein in GPRC6A-HEK293A cells as a band of 105 kDa. (B) Increases of the basal pERK/ERK levels after stimulation with 20 and 30 mM L-arginine of GPRC6A-HEK293A cells transiently transfected with different amount of GPRC6A plasmid (2 and 4 μg). (C) Densitometric analysis of the western blot shown in b. (D) Western blot detection of CASR protein in CASR-HEK293A cells as two bands of 100 and 150 kDa. (E) Significant increases of the basal pERK/ERK levels after stimulation of CASR-HEK293A cells with increasing concentrations of [Ca2+]o (1.0, 3.0 and 5.0 mM). (F) Densitometric analysis of the western blot shown in e. (G) Basal pERK/ERK level increases after stimulation with different concentrations (50 and 100 nM, 0.5 and 5.0 μM) of the potent CASR agonist R568 (Cayman Chemical Company; Ann Arbor, Michigan, USA). (H) Densitometric analysis of the western blot shown in g. [file Image_1.tif]

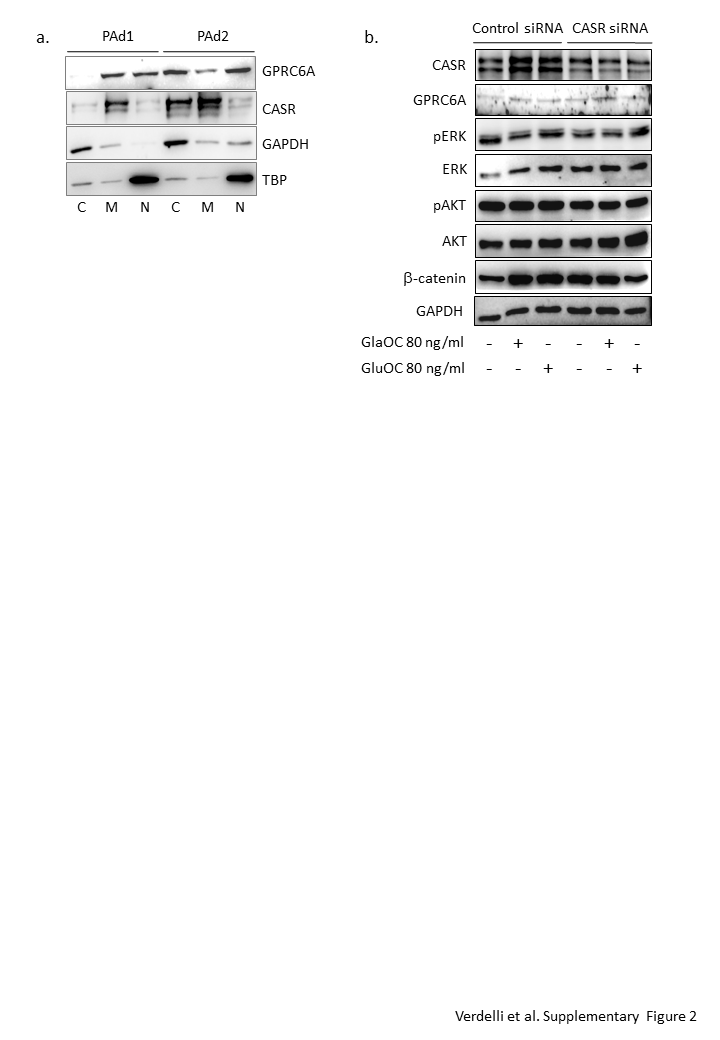

Supplement: Supplementary Figure 2 — (A) Representative western blot analysis of the GPRC6A and CASR proteins’ expression in membrane protein fractions from 2 PAds (TBP ab51841, Abcam, Cambridge, UK). (B) Exemplificative western blots obtained in a GPRC6A-expressing PAd transfected with Control siRNA and with CASR siRNA. [file Image_2.tif]
