# Supplementary material for: Osteocalcin modulates parathyroid cell function in human parathyroid tumors
Source: Front Endocrinol (Lausanne). 2023 Mar 30;14:1129930. doi: 10.3389/fendo.2023.1129930 (PMC10098338; doi:10.3389/fendo.2023.1129930)
Supplement: Supplementary Table 1 — Clinical and biochemical features of PHPT patients, whose surgically removed PAds were analyzed for CASR and GPRC6A membrane expression. Patient ID, identification corresponding to that used in; age, PHPT patient age at diagnosis; BMI, body mass index; Ca2+, plasma ionized calcium; SCa, serum total calcium; PTH, plasma parathormone; creat, serum creatinine; tumor size, maximal diameter of the surgically removed parathyroid adenoma; kidney, diagnosis of kidney stones symptomatic disease and asymptomatic stones detected by imaging); bone, diagnosis of osteopenia/osteoporosis according WHO criteria (49). [file Table_1.docx]

**Supplementary Table 1.** Clinical and biochemical features of PHPT patients, whose surgically removed PAds were analyzed for CASR and GPRC6A membrane expression.

| **Patient** | **Sex** | **Age** | **BMI** | **Ca^2+^** | **SCa** | **PTH** | **Creat** | **Tumor Size** | **Kidney** | **Bone** |
| --- | --- | --- | --- | --- | --- | --- | --- | --- | --- | --- |
| ID |  | years | kg/m^2^ | mmol/L | mg/dl | pg/ml | mg/dl | cm |  |  |
| PAd1 | F | 57 | 28.5 | 1.48 | 11.3 | 127 | 0.70 | 2.2 | No | Yes |
| PAd2 | F | 50 | 27.0 | 1.78 | 12.3 | 231 | 0.81 | 1.5 | No | Yes |
| PAd3 | M | 50 | 23.2 | 1.74 | 13.0 | 231 | 1.25 | 1.2 | No | Yes |
| PAd4 | M | 48 | 25.7 | 1.73 | 12.9 | 367 | 0.83 | 2.2 | Yes | No |
| PAd5 | M | 72 | 25.6 | 2.97 | 22.4 | 1694 | 1.01 | 2.5 | No | Yes |
| PAd6 | F | 73 | 21.1 | 1.48 | 11.9 | 166 | 0.56 | 1.0 | No | Yes |
| PAd7 | F | 68 | 21.9 | 1.81 | 14.0 | 662 | 1.00 | 3.7 | No | Yes |
| PAd8 | F | 45 | 26.9 | 1.41 | 12.5 | 112 | 0.78 | 1.6 | Yes | No |
| PAd9 | F | 71 | 26.7 | 1.52 | 13.9 | 368 | 1.09 | 2.0 | No | Yes |
| PAd10 | M | 59 | 28.1 | 1.54 | 11.5 | 116 | 0.93 | 1.4 | No | No |
| PAd11 | F | 55 | 28.1 | 1.38 | 10.6 | 105 | 0.70 | 2.0 | No | Yes |
| PAd12 | F | 77 | 27.5 | 1.27 | 9.5 | 114 | 1.03 | 1.1 | No | Yes |
| PAd13 | F | 57 | 34.9 | 1.56 | 12.1 | 192 | 0.66 | 1.4 | No | No |
| PAd14 | F | 44 | 27.7 | 1.52 | 12.2 | 628 | 0.79 | 1.1 | No | No |
| PAd15 | F | 63 | 18.6 | 1.49 | 11.1 | 147 | 0.73 | 0.8 | No | Yes |

Patient ID, identification corresponding to that used in Figure 4; age, PHPT patient age at diagnosis; BMI, body mass index; Ca^2+^, plasma ionized calcium; SCa, serum total calcium; PTH, plasma parathormone; creat, serum creatinine; tumor size, maximal diameter of the surgically removed parathyroid adenoma; kidney, diagnosis of kidney stones symptomatic disease and asymptomatic stones detected by imaging); bone, diagnosis of osteopenia/osteoporosis according WHO criteria(49).
